# Complete talectomy for post-traumatic osteomyelitis (and/or avascular necrosis): report of a new technique

**DOI:** 10.5194/jbji-10-419-2025

**Published:** 2025-10-30

**Authors:** Daniel Pérez-Prieto, Alois Baumer, Jan Martinez-Lozano, Julian Aquilina, Paul Zamora, Albert Alier, Lluïsa Sorlí

**Affiliations:** 1 Septic Unit, Orthopedic Surgery Department, Hospital del Mar, Passeig Marítim de la Barceloneta 25–27, 08003 Barcelona, Spain; 2 IcatRecon, Hospital Dexeus, Barcelona, Carrer Sabino Arana, 5–19, 080029 Barcelona, Spain; 3 Department of Surgery, St Mary's Hospital, Isle of Wight, United Kingdom; 4 Plastic Surgery Department. Hospital del Mar, Passeig Marítim de la Barceloneta 25–27, 08003 Barcelona, Spain; 5 Infectious Disease Department, Hospital del Mar, Passeig Marítim de la Barceloneta 25–27, 08003 Barcelona, Spain; 6 Septic Diseases Department, Hospital del Mar, Passeig Marítim de la Barceloneta 25–27, 08003 Barcelona, Spain

## Abstract

The main complications after complex talar fractures, especially with respect to open injuries, are avascular necrosis (AVN) and fracture-related infection (FRI) Their treatment is a source of discussion, and reconstruction options are scarce.

A descriptive longitudinal study of three cases with a two-stage tibiocalcaneal (TC) arthrodesis (talectomy followed by a retrograde nailing and two tantalum spacers) is presented.

Information on infection relapse and fusion of the arthrodesis was collected, along with demographic, radiological, and functional variables (such as Manchester–Oxford Foot Questionnaire, MOXFQ, values; EuroQol index values; and visual analogue scale for pain, VAS-pain, values)

After a minimum of 3 years, no infection relapse or pseudoarthrosis was observed. Leg alignment was comparable to the contralateral side. Functional and pain tests showed optimal values: MOXFQ index of 16.6, mean EuroQol index of 0.782, and mean VAS-pain of 19.

For a salvage procedure in FRI + AVN of the talus, this two-stage TC arthrodesis is a safe procedure in terms of infection and provides good functional outcomes.

## 1 Introduction

Complex fracture–dislocations of the talus, although uncommon, are frequently related to complications (Hamilton et al., 2024; Haskell, 2019; Salem, 2008). Avascular necrosis (AVN) is one of the most frequent complications and one of the most challenging to manage (Backus and Ocel, 2019). The frequency of talar AVN associated with talar fractures has been reported to be approximately 10 %, 40 %, and 90 % in Hawkins types 1, 2, and 3, respectively (DiGiovanni et al., 2007; Haskell, 2019). Fracture-related infection (FRI) after fixation is reported with a likely underestimated rate of 12 % and, together with avascular necrosis (AVN) and osteomyelitis (OM), is a particularly difficult complication to treat (Weston et al., 2015; Metsemakers et al., 2018, 2020). Regarding open fractures and dislocations of the talus, it is not uncommon for AVN and FRI to occur together. There are a few case reports in the literature that discuss these complications using different approaches (Kolker and Wilson, 2004; LaPorta et al., 2014; Stapleton and Zgonis, 2013).

The purpose of the present study is to describe three cases of AVN and/or FRI treated by means of a two-stage procedure (Masquelet-like technique) and tibiotalocalcaneal arthrodesis (TTCA). The surgical technique and the clinical and radiological outcomes at a minimum follow-up of 2 years are presented (Tong et al., 2017).

## 2 Methods

### 2.1 Surgical technique

A two-stage procedure was done in all three cases. In the first surgery, a complete talectomy and a thorough debridement and lavage was performed. Five specimens were obtained for microbiological assessment, while two specimens were obtained for anatomopathological evaluation (Metsemakers et al., 2018; Govaert et al., 2020). According to previous cultures (when they exist), commercially available antibiotic-loaded bone cement (ALBC) was used with clindamycin plus gentamicin or vancomycin plus gentamicin (COPAL C + G^®^ or COPAL V + G; Heraeus. Wehrheim, Germany) (Metsemakers et al., 2020; Hellebrekers et al., 2019). Moreover, manually added vancomycin, up to 4–6 g per 40 g of ALBC, was considered in all cases.

**Figure 1:**
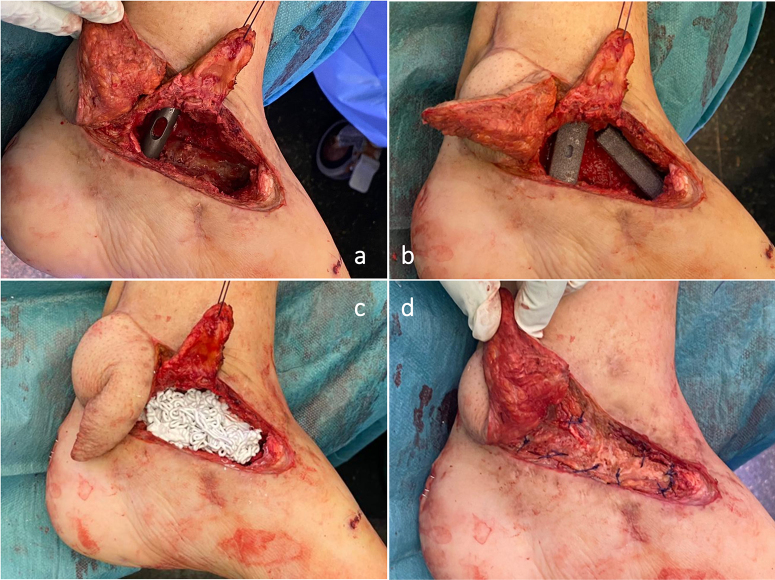
Intraoperative images. In this composed image, the following are presented: the arthrodesis nail **(a)**, tantalum spacers in the void left by the talus **(b)**, the remaining void filled with CERAMENT^®^ **(c)**, and the autograft and closure of the Masquelet membrane **(d)**.

After 6–8 weeks, the second stage was scheduled. In the first step, an autograft from the ipsilateral femur was obtained by means of a reamer–irrigator–aspirator (RIA^®^, DePuy Synthes, J&J Medtech) system. The autograft was soaked in gauze with a 5 mg mL^−1^ vancomycin solution (Pérez-Prieto et al., 2016). Afterwards, the ALBC spacer was removed, the Masquelet membrane was preserved, and five specimens for microbiological assessment and two for anatomopathological evaluation were obtained (Hellebrekers et al., 2019). A tibiotalocalcaneal (TTC) arthrodesis was performed using a T2^®^ ankle arthrodesis nail (Stryker. Schönkirchen, Germany). Two tantalum spacers were employed (Zimmer-Biomet Spine, Westminster, Colorado, USA) (Cohen and Kazak, 2015): one from the anterodistal tibia to the navicular bone and one from the distal tibia to the calcaneus. Three columns of axial stability were achieved this way: the fibula on the lateral side, a nail in the middle, and a tantalum spacer on the medial side (Fig. 1).

The void was filled with CERAMENT^®^ G and CERAMENT^®^ V (BONESUPPORT, Lund, Sweden) using a *sandwich technique* along with the autograft obtained from the femur (Fig. 1) (Bezstarosti et al., 2021).

The wound was closed in layers (including the Masquelet membrane), and the patient avoided weight-bearing activity for 4 weeks. From the fifth week to the eighth week, progressive weight-bearing activity with a walker boot was allowed.

### 2.2 Case presentations

#### 2.2.1 Case 1

A 64-year-old male was seen after a below-the-knee amputation was recommended elsewhere due to failure of infection resolution and with diagnosed AVN and FRI of the left talus (Metsemakers et al., 2020, 2018).

In August 2019, the patient suffered an open dislocation of the talus without fracture. It was reduced and fixed with 2 K-wires. One week later, he underwent reoperation due to purulent discharge; the infection was not controlled and skin necrosis appeared. A fasciocutaneous anterolateral free flap of the thigh associated with a new debridement was performed 1 month later.

**Figure 2:**
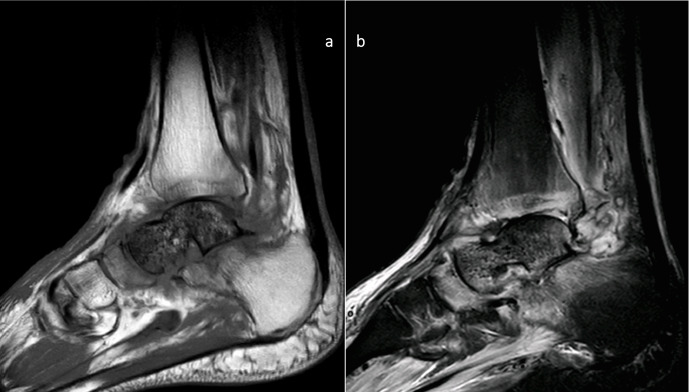
MRI images of the preoperative state of the talus. Sagittal MRI images showing signs of avascular necrosis and osteomyelitis: **(a)** T1 and **(b)** STIR images.

In November 2019, the infection relapsed; at this point, he was referred to our hospital. The flap was in a good condition, with some erythema around it. MRI confirmed AVN and FRI with no other bones involved or purulent collections (Fig. 2).

In this case, according to previous cultures (polymicrobial, including multidrug-resistant Gram-negative bacilli), manually added meropenem (plus vancomycin, as previously mentioned) to the COPAL C + G^®^ ALBC was used in the first stage. Due to the partial stability of the construct, a temporary external fixator was used.

Pathology confirmed OM, and cultures were positive to *Serratia marcescens* (quinolone-resistant), *Enterococcus faecalis* and *Actinomyces odontolyticus*. Given the lack of oral antibiotic treatment, the patient was maintained on an intravenous antibiotic regime (ampicillin + imipenem) for 8 weeks.

Cultures of the second stage and the pathology evaluation were all negative; therefore, antibiotics were stopped after 2 weeks of this stage.

#### 2.2.2 Case 2

A 43-year-old male was seen due to failure to control infection and with the diagnosis of AVN and FRI of the right talus.

In May 2019, he suffered a closed right talus fracture–dislocation (Hawkins type 4) after a traffic accident. It was reduced and fixed with two screws and an external fixator. One month later, he underwent reoperation due to purulent discharge; the infection was not controlled. An anterolateral free flap associated with a new debridement was performed 1 month later.

In October 2020, he was referred to our hospital. The flap was in a correct status, and MRI confirmed AVN and FRI of the talus.

In this case, according to previous cultures (*Staphylococcus aureus*), an ALBC of COPAL C + G^®^ plus manually added vancomycin was used in the first stage.

Pathology confirmed OM, with cultures indicating a multidrug-resistant *Pseudomonas aeruginosa*. The patient underwent an 8-week course of intravenous antibiotics, consisting of ceftazidime/avibactam, colistin, and daptomycin.

Cultures of the second stage and the pathology evaluation were normal; therefore, antibiotics were discontinued after 2 weeks.

#### 2.2.3 Case 3

A 22-year-old male was seen after a talectomy due to FRI of the right talus performed in another country.

In November 2021, the patient suffered a hematoma in the right ankle after a sports accident that became infected 2 weeks later with purulent discharge.

An MRI at the treating centre revealed OM without AVN. A new debridement and the first stage of the surgery (complete talectomy) were performed using local antibiotics and a temporary external fixator to ensure the stability of the construct. A fasciocutaneous flap was also performed at this point.

Cultures were positive for *Bacillus* species, *Enterobacter cloacae*, and *Enterobacter asburiae*. The patient was treated with three different antibiotic regimens for 6 weeks (piperacillin/tazobactam and vancomycin, teicoplanin, and ciprofloxacin and dalbavancin).

He was referred to hospital in January 2022 for the second stage of surgery in February. Cultures of the second stage and the pathology evaluation were negative; therefore, antibiotics were stopped after 2 weeks.

## 3 Results

No recurrence of infection was observed in any case with a follow-up of 60, 56, and 36 months. All of the patients returned to work and resumed the activities of daily living.

**Figure 3:**
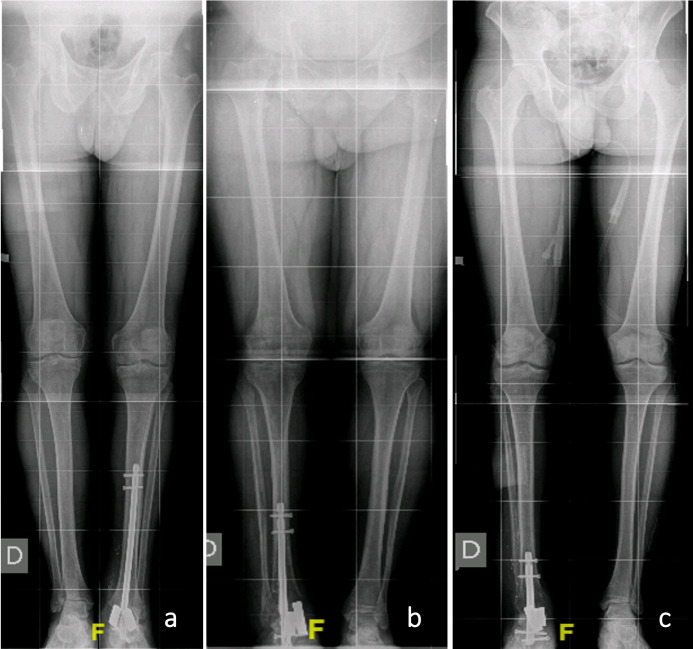
Postoperative alignment. Standing long-leg images corresponding to cases 1 **(a)**, 2 **(b)**, and 3 **(c)**, respectively

**Figure 4:**
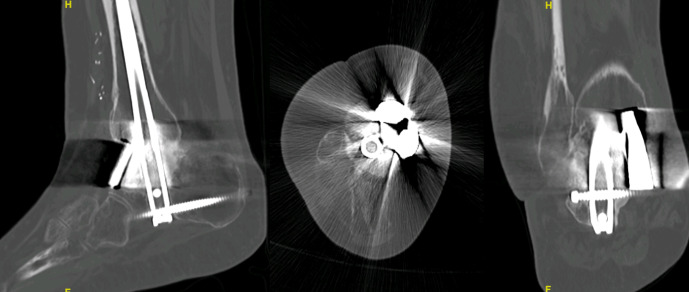
In spite of the image distortion due to radiolucent osteosynthetic material, signs of consolidation and the absence of indirect signs of pseudoarthrosis can be noted in the 1-year control CT scan of one of the patients (Case 2). This image, along with a painless weight-bearing ambulation, is enough to consider a consolidation of the defect.

Total fusion was seen in the 1-year follow-up CT scan control, and a correct axis may be seen in X-rays, with no significative leg-length discrepancy (Figs. 3 and 4).

Functional outcomes, measured using the Manchester–Oxford Foot Questionnaire, and quality of life outcomes, measured using the EuroQol index, may be seen in Table 1.

**Table 1:** Clinical scores obtained at the end of follow-up period.

	Age	Follow-up	EuroQol	EuroQol	VAS-pain	MOXFQ			
		(months)	index	VAS		Index	Pain	Walk	Social
Patient 1, male	64	60	0.887	77	17	15.2	15	19.4	8.5
Patient 2, male	43	56	0.661	68	30	22.9	35	20.8	10.8
Patient 3, male	22	35	0.799	95	10	6.1	0	12.1	5
Average	43	50.3	0.782	80	19	14.7	16.6	17.4	8.1

VAS represents the visual analogue scale. MOXFQ refers to the Manchester–Oxford Foot Questionnaire.

## 4 Discussion

Although AVN and OM of the talus constitute a serious and potentially limb-threatening complication, good functional outcomes and an infection-free period of at least 3 years are achieved with the current technique (Haskell, 2019). The two-stage approach, with a TTC ankle arthrodesis with tantalum augmentation and void filling with CERAMENT^®^, is a reproducible and safe technique that allows early weigh-bearing activity with no leg-length discrepancy. Soft tissues must be in a correct status; otherwise, a free flap is mandatory.

AVN of the talus often requires surgical treatment, especially when it presents after a fracture. Many of the cases are associated with osteoarthritis, and TTCA is the most frequent treatment. Using new 3-D bio-printing techniques, some authors have reported good outcomes related to performing a TTCA with a custom-made metal bone defect (Grau et al., 2022; Ramhamadany et al., 2021; Shnol and LaPorta, 2018) Similarly, talus prosthesis has been described as an option; however, this technique is associated with a high rate of complications such as arthrofibrosis, dislocation, and subtalar osteoarthritis (Mauren et al., 2021; Taniguchi et al., 2015; Jennison et al., 2023).

Regarding FRI of the talus, few data are available. However, it is well known that when there is an infection in a necrotic bone, there is no possibility of retaining this bone to cure the infection (Metsemakers et al., 2018, 2020). In that sense, all of the case reports found in the literature involved a complete talectomy. Nevertheless, they differ with respect to the reconstructive solution employed. Direct tibiocalcaneal arthrodesis is a safe technique, although it induces an important leg-length discrepancy (Kolker and Wilson, 2004; Stapleton and Zgonis, 2013). However, it may be useful when soft tissues are at risk and a free flap is not possible. In these cases, it is not suitable for young patients.

Talus prosthesis has also been described in a case of OM and AVN. The authors reported good outcomes using a short follow-up period. This is an attractive technique, but the complications mentioned above must be taken into account. Moreover, in cases of infection, a good antibiofilm antibiotic should be available (Zimmerli et al., 2004; Pérez-Prieto et al., 2019).

The case reports using a TTC two-stage approach have some differences compared with the cases presented here (LaPorta et al., 2014). The use of tantalum augmentation provides a stronger construct. A complete talectomy jeopardizes the talonavicular joint and provokes instability in this part of the midfoot (Cohen and Kazak, 2015). The tantalum spacer acts as “talar neck” in that sense. Moreover, the medial vertical tantalum spacer provides a medial column stability, as previously mentioned. Another difference is the use of a calcium sulfate plus hydroxyapatite and antibiotic (CERAMENT^®^, BONESUPPORT, Lund, Sweden) as the void filler (Ferguson et al., 2019; Metsemakers et al., 2019). It is clear that a bone autograft is the best option for arthrodesis. However, the volume that one can obtain is limited, and the eventual residual bacteria in the second stage supports the use of CERAMENT^®^, as it provides bone remodelling plus local antibiotics (Bezstarosti et al., 2021). Finally, the most important difference is that the present study shows the longest follow-up period reported: up to 5 years without further complications.

Nevertheless, the present study has some limitations (apart from the limited number of cases). Although it is stated that 2 years free of infection is an adequate follow-up, full cure cannot be fully assured (Metsemakers et al., 2018, 2020). Degenerative processes in the surrounding joints may provide future issues, especially for younger patients, and the superiority of other techniques, such as talus prosthesis, may be possible in these situations.

## 5 Conclusions

A two-stage approach after AVN and/or OM is a safe procedure in terms of infection and provides good functional outcomes with no leg-length discrepancy. This two-stage approach consists of a complete talectomy, a Masquelet-like technique and a TTCA, and the use of synthetic bone graft substitute with antibiotics and tantalum struts.

## Data Availability

The research data have not been made publicly available online due to patient requests. However, the data can be shared upon reasonable request by contacting the corresponding author.
